# The Elicitation of an Antigen-Specific Antibody Immune Response Using a Nanoparticulate Adjuvant Derived from *Saponaria officinalis*

**DOI:** 10.3390/molecules30163328

**Published:** 2025-08-09

**Authors:** Andrey Bogoyavlenskiy, Madina Alexyuk, Pavel Alexyuk, Elmira Omirtayeva, Irina Zaitseva, Yergali Moldakhanov, Elmira Anarkulova, Vladimir Berezin

**Affiliations:** Research and Production Center for Microbiology and Virology, 105, Bogenbai Batyr Street, Almaty 050010, Kazakhstan

**Keywords:** saponin, *Saponaria officinalis*, vaccine, adjuvant, viral antigen, specific immunity

## Abstract

The use of vaccines incorporating subunit proteins and viral components has significantly increased in recent decades, emphasizing the need for more effective and modular adjuvants. This study examined saponins from *Saponaria officinalis*, regarded as one of the most promising plant sources for developing an adjuvant platform using nanocomplex formation. A nanoparticle adjuvant containing saponins from *Saponaria officinalis* can be used to stimulate a humoral immune response; this ability was demonstrated using a model that included various viral proteins. The humoral immune response enhanced by saponin-containing adjuvants can increase from four to sixteen times, depending on the type of antigen used. Additionally, this response surpasses that triggered by antigens paired with aluminum hydroxide and is comparable to responses induced by adjuvants that contain Quil A. The further investigation of these platforms may yield a broader range of immunostimulants that can enhance vaccine effectiveness.

## 1. Introduction

The early years of the third millennium have seen a significant increase in infectious diseases affecting both humans and animals. The global economic impact of these diseases is estimated to reach several trillion dollars each year. Notably, the economic losses from influenza and acute respiratory infections account for at least 50% of the total economic damages caused by infectious diseases in humans [1,2].

To date, vaccination remains the most effective method for combating infectious diseases. The current research aimed at enhancing the quality and effectiveness of vaccines has taken two main directions. The first direction focuses on creating new types of vaccines such as split vaccines, subunit vaccines, RNA or DNA vaccines, and vaccines developed using strains obtained through reverse genetics. The second direction involves developing new adjuvants that can boost protective immunity without requiring a higher antigen dose in the vaccine. This also allows for effective vaccination in individuals with compromised immune systems. Among the most promising new-generation adjuvants are nanoparticle platforms containing triterpene saponins [3,4,5]. This platform leverages the ability of saponins to interact with cholesterol and phospholipids, resulting in the formation of specific supramolecular complexes with immunostimulating properties [6,7].

Most studies on plant-origin immunostimulants were carried out with the saponin-containing preparation Quil A, derived from the bark of the South American tree *Quillaja saponaria Molina*. As shown in numerous studies, the saponins contained in this preparation can form multimolecular complexes with lipids and antigens of various origins. Immunostimulatory complexes based on saponins isolated from the bark of *Q. saponaria Molina* were created using many viral, bacterial, and parasitic antigens [8,9,10]. It was established that the basis of the immunostimulatory complex is a matrix that is a stable spatial structure containing saponins from *Q. saponaria* [8,9,10].

It has been established that this immunostimulating matrix possesses immunostimulating properties without the need for preliminary protein inclusion [11,12].

The development of immunostimulating complexes (matrix M) with less toxicological effects and the potential for chemical synthesis of saponins has helped us partially resolve these issues [10]. The Matrix-M™ (Isconova AB, Uppsala, Sweden) adjuvant is included in several candidate vaccines in the clinical developmental phase including targeting seasonal influenza (alone and in combination with COVID-19), malaria, and Ebola Virus Disease [13,14,15,16,17].

Nonetheless, further research is needed in this area. Only a handful of adjuvants are currently clinically licensed for use in vaccine formulations and approved by the Food and Drug Administration (FDA). These include more traditional insoluble aluminum salts (Alum) and oil-in-water emulsions such as MF59 and AS03 [18]. Recent FDA approvals of vaccines comprising CpG-ODN 1018 (Dynavax), AS04 (GSK; MPL/Alum), and AS01 (GSK; liposomes comprising MPL), along with numerous ongoing clinical trials using other promising TLRas such as 3M-052 (3M/AAHI), reinforce their promise as adjuvants to improve the efficacy of vaccines [19].

As preliminary studies have shown, among Kazakhstan’s flora, many plants contain triterpene saponins, which can significantly enhance the activity of the immune response and the effectiveness of vaccines. Triterpene saponins isolated from Kazakhstani plants can serve as organizers of highly immunogenic immunostimulatory nanocomplexes when interacting with lipids and protein antigens of various origins [20,21,22]. This research aimed to study the ability of the nanocomplexes with saponins from *Saponaria officinalis* to stimulate a humoral specific immune response to viral antigens.

## 2. Results

### 2.1. Characterization of Saponin-Containing Complexes

**Figure 1:**
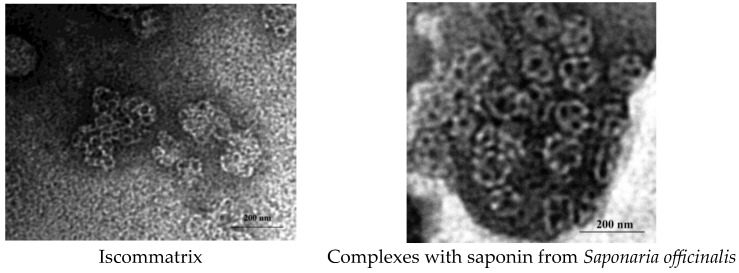
Supramolecular saponins complexes. The structure and sizes of the nanocomplexes were observed via electron microscopy using negative staining and an instrumental magnification of 1:100,000.

The mean diameter and charge of formulations were calculated with a zeta sizer (Malvern, UK), and the results are presented in Table 1. The polydispersity indexes of complexes with QuilA and saponins from *Saponaria officinalis* were 0.21 and 0.30, respectively; this indicates that all different formulations are fairly homogenous. The zeta potential of all preparations was negative due to the presence of Quil A or saponins from *Saponaria officinalis* (Table 1). The Encapsulation Efficiency values were 92 and 94%, respectively (Table 1). The size of the supramolecular structures was in the range of 40–80 nm as measured via transmission electron microscopy (Figure 1). TEM was used to confirm both the sizes as well as the morphology of the colloids; the morphology showed hollow cage-like structures, which are considered the typical morphology of ISCOMATRIX.

Table 2 presents the results of the toxicity of the studied saponins on cell cultures. The saponin of *Saponaria officinalis* was shown to have the ability to cause the death of 50% of cells at a dose eight times higher than Quil A. The formation of supramolecular complexes reduces the toxicity of both drugs, but the saponin of *Saponaria officinalis* retains less pronounced toxicity.

### 2.2. Induction of an Antigen-Specific Antibody Immune Response Using Influenza Virus Glycoprotein Antigens Combined with Adjuvants

A comparative study was performed on the ability of supramolecular complexes to stimulate humoral immunity using a model of purified influenza virus glycoproteins with various antigen formulations. The dose of viral antigens was 10 μg per animal and that of saponin was 15 μg per animal. A placebo comprising iscommatrix with Quil A and aluminum hydroxide was used as a control. The immunostimulating activity of saponins without complex formation was also compared.

**Figure 2:**
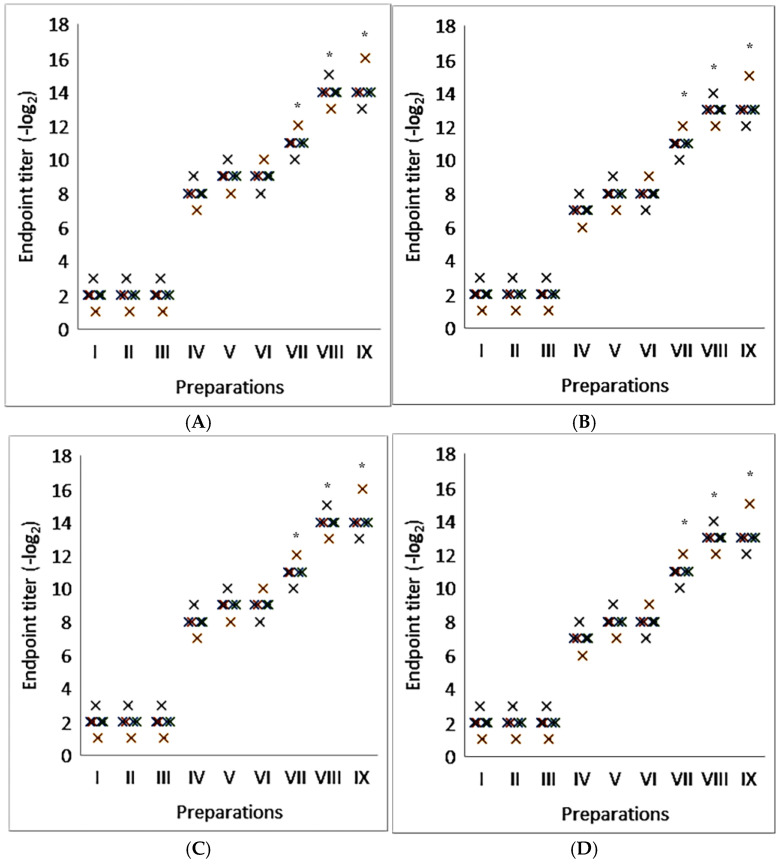
Immunostimulatory activity of nanocapsulated adjuvant with saponins from *Saponaria officinalis* in model of viral glycoproteins. I—placebo; II—Quil A; III—saponin from *Saponaria officinalis*; IV—viral glycoproteins; V—viral glycoproteins with aluminum hydroxide; VI—viral glycoproteins with Quil A; VII—viral glycoproteins with saponin from *Saponaria officinalis*; VIII—viral glycoproteins with iscommatrix with Quil A; IX—viral glycoproteins with supramolecular complexes with saponin from *Saponaria officinalis*. (**A**)—human influenza virus strain A/Almaty/8/98 (H3N2); (**B**)—avian influenza virus strain A/Tern/South Africa/1/1961 (H5N3); (**C**)—highly pathogenic avian influenza virus strain A/FPV/Rostock/34 (H7N1); (**D**)—Tamiflu-resistant pandemic variant A/Vladivostok/2/09 (H1N1). The results are the average results from five separate mice. * Statistically significant difference (*p* < 0.05) compared to viral glycoproteins.

As shown in Figure 2, regardless of the type of glycoprotein antigens used, the titer of humoral immunity increased from eight to sixteen times when using complexes containing the saponin of *Saponaria officinalis* compared to purified proteins. At the same time, the level of antibodies exceeded the activity of that when using aluminum hydroxide and was comparable with complexes containing Quill A. It was established that saponins, without the formation of complexes, can stimulate the formation of humoral immunity that does not exceed the control by more than two times.

**Figure 3:**
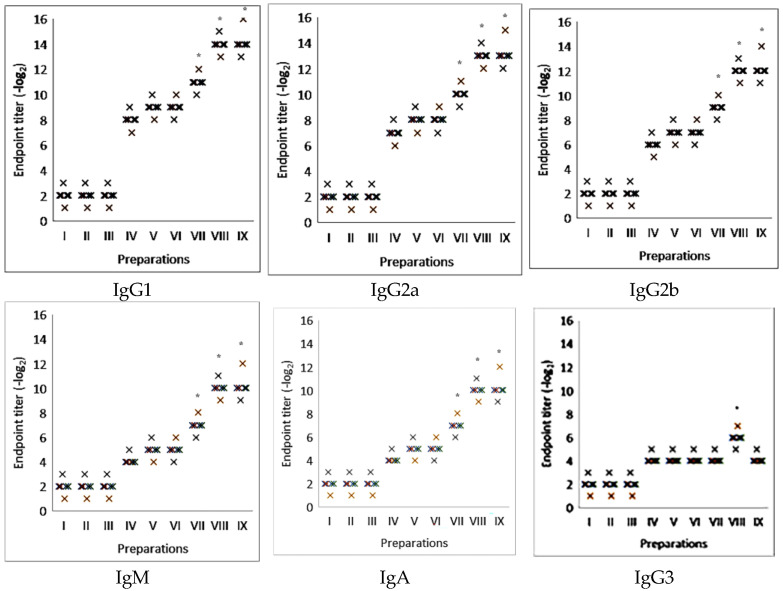
Immunostimulatory activity of different IgG classes with the nanocapsulated adjuvant with saponins from *Saponaria officinalis* in the model of viral glycoproteins. I—placebo; II—Quil A; III—saponin from *Saponaria officinalis*; IV—viral glycoproteins; V—viral glycoproteins with aluminum hydroxide; VI—viral glycoproteins with Quil A; VII—viral glycoproteins with saponin from *Saponaria officinalis*; VIII—viral glycoproteins with iscommatrix with Quil A; IX—viral glycoproteins with supramolecular complexes with saponin from *Saponaria officinalis*. The results are the average of five separate mice. * Statistically significant difference (*p* < 0.05) compared to viral glycoproteins.

Further studies on the H3N2 influenza virus model examined the ability of various complexes to stimulate different classes of immunoglobulins (Figure 3). The findings demonstrated that these complexes can stimulate not only immunoglobulin classes M and A but also subclasses IgG1, IgG2a, and IgG2b. Additionally, unlike complexes containing Quil A, saponin complexes did not promote the stimulation of IgG3, indicating that they are less reactogenic.

Thus, complexes including saponin from Saponaria officinalis stimulate the formation of humoral immunity to glycoproteins of the influenza virus with different antigen structures, including not only the main classes IgG, IgM, and IgA but also subclasses IgG2a and IgG2b. This indicates the possibility of stimulating not only humoral but also cellular immunity. In this case, the level of immunity exceeded that when using the standard adjuvant aluminum hydroxide by at least eight times, comparable with the immunogenicity of the iscommatrix containing Quill A.

### 2.3. The Effectiveness of Antigen-Specific Antibody Immune Response Induction Using Nanoparticulate Adjuvant from Saponaria officinalis Saponin in the Model of Commercial Viral Antigens

The effectiveness of inducing an antigen-specific antibody immune response using a nanoparticulate adjuvant containing saponin from *Saponaria officinalis* was studied in a model of genetically engineered Dengue virus envelope protein containing antigenic determinants of the 1 and 4 virus types, herpes simplex virus-1 recombinant protein, and West Nile virus envelope recombinant protein. The dose of viral antigens was 10 μg per animal and that of saponin was 15 μg per animal. A placebo comprising iscommatrix with Quil A and aluminum hydroxide was used as a control. The immunostimulating activity of saponins without complex formation was also compared.

**Figure 4:**
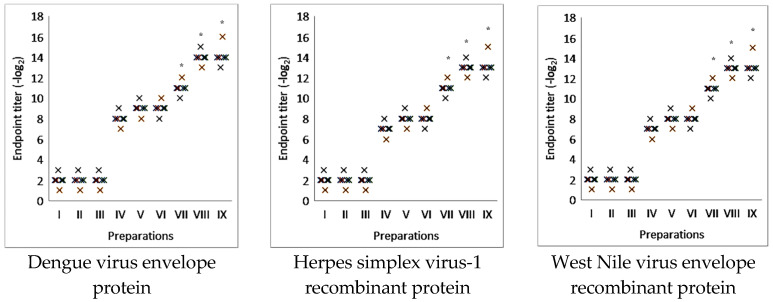
Immunostimulatory activity of nanocapsulated adjuvant from *Saponaria officinalis* in model of viral proteins: Dengue virus envelope protein, HSV-1 recombinant protein, and West Nile virus envelope protein. I—placebo; II—Quil A; III—saponin from *Saponaria officinalis*; IV—viral glycoproteins; V—viral glycoproteins with aluminum hydroxide; VI—viral glycoproteins with Quil A; VII—viral glycoproteins with saponin from *Saponaria officinalis*; VIII—viral glycoproteins with iscommatrix with Quil A; IX—viral glycoproteins with supramolecular complexes with saponin from *Saponaria officinalis.* The results are the average of five separate mice. * Statistically significant difference (*p* < 0.05) compared to viral antigen.

Research has shown that complexes containing saponin from *Saponaria officinalis* can effectively increase the level of IgG by four to eight times, depending on the specific type of genetically engineered protein used. Furthermore, the immunostimulating activity of these complexes was found to be greater than that of aluminum hydroxide and comparable to the immunostimulating activity of iscommatrix, which contains Quil A (Figure 4).

Complexes containing Saponaria officinalis saponin can stimulate the humoral immune response to various viral antigens including genetically engineered ones.

### 2.4. Induction of Virus Neutralization Immune Response with Influenza Virus Glycoprotein Antigens or Purified Whole Influenza Virions Mixed with Adjuvants

**Figure 5:**
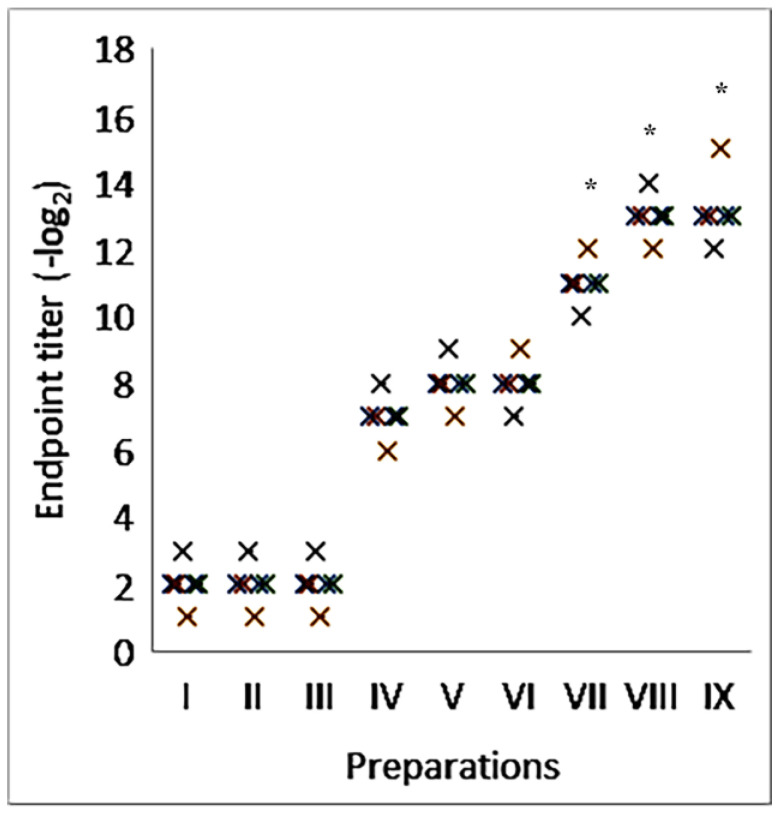
Induction of virus neutralization immune response. I—placebo; II—Quil A; III—saponin from *Saponaria officinalis*; IV—viral glycoproteins; V—viral glycoproteins with aluminum hydroxide; VI—viral glycoproteins with Quil A; VII—viral glycoproteins with saponin from *Saponaria officinalis*; VIII—viral glycoproteins with iscommatrix with Quil A; IX—viral glycoproteins with supramolecular complexes with saponin from *Saponaria officinalis*. * Statistically significant difference (*p* ≤ 0.05) compared to viral glycoproteins.

The induction of a virus-neutralizing immune response was studied after animal immunization with influenza virus glycoproteins in combination with various adjuvants. The experiments were carried out with glycoproteins of influenza virus type A: H3N2. The immunization of mice was shown to stimulate the formation of virus-neutralizing antibodies, the level of which was comparable to that of a specific immune response (Figure 5).

Thus, the studied supramolecular complexes can stimulate both humoral and virus-neutralizing immune responses.

## 3. Discussion

In today’s rapidly evolving field of vaccinology, research has increasingly focused on developing new adjuvants. These adjuvants enhance vaccine effectiveness, utilizing nanoparticle delivery systems for the administration of antigens. This innovative approach aims to improve the overall efficacy of vaccines [23,24,25,26].

Nanoparticles with immunostimulating properties, including inorganic, lipid, and polymer nanoparticles, as well as microemulsions, can regulate immune cells and their signaling pathways while also exerting their immunomodulatory effects. This highlights the dual role of nanoparticles in adjuvant platforms.

However, testing the immunostimulating activity of these systems in vitro—outside living organisms—can be complex, and various side effects may arise. This has led researchers to seek characteristics that indirectly indicate the presence of immunostimulating activity in nanoparticles. Key properties include the shape, size, and zeta potential values of the nanoparticles.

Research has shown that for nanoparticles to exhibit immunostimulating activity, they must meet certain criteria: they should be spherical, not exceed 300 nm in size, and have a zeta potential ranging from −30 to +30 mV [27,28,29,30]. These conditions are predominantly satisfied by platforms like ISCOMMATRIX™ and MATRIX M (Isconova AB, Uppsala, Sweden) [10,11,12,17,18,19]. The ISCOMMATRIX™ adjuvant enhances vaccine efficacy by acting as both an antigen delivery vehicle and a potent immunomodulator, leading to strong humoral and cellular immune responses. It works by interacting with immune cells like dendritic cells, promoting efficient antigen presentation and enhancing the expression of co-stimulatory molecules [10,12].

The creation of such platforms is based on the ability of triterpene saponins in aqueous solutions to self-organize into various supramolecular structures in the presence of cholesterol and phospholipid molecules [31,32,33]. This property was utilized to create the vaccine adjuvant Quil A, which is capable of stimulating all aspects of the immune response. Subsequent research revealed that the protein-free complex, referred to as the iscommatrix, and matrix M exhibited the same properties. This discovery laid the groundwork for creating an immunostimulating platform that generates supramolecular immunostimulating structures. Consequently, several new vaccines for both livestock and human medicine have been developed using this adjuvant platform, targeting various viral, parasitic, and microbial antigens. Clinical trials have been completed for several of these vaccines [12].

Building on the successful experience with Quil A [34,35,36], numerous research efforts have emerged focused on discovering new sources of saponins for developing similar adjuvant platforms. Studies have demonstrated that saponins with various structures and origins possess immunostimulating properties [37,38,39]. Triterpene compounds from various plant families can form supramolecular complexes with immunostimulating properties [37,38,39]. One promising source of these compounds is the Caryophyllaceae plant family, which contains quillaic acid and can form immunostimulating complexes with proteins from different sources [20,21,22].

Our studies have shown that saponin from Saponaria officinalis is capable of forming supramolecular complexes with phospholipids and cholesterol, the physical properties of which correspond to immunostimulating activity: a particle size less than 100 nm, zeta potential of −15.8 mv, and honeycomb-like structures.

In further studies, we examined the ability of saponin complexes from Saponaria officinalis to stimulate the formation of humoral immunity against various viral antigens of epidemiological significance.

Studying the formation of the humoral immune response using influenza virus glycoproteins with varying antigen structures and genetically engineered viral proteins revealed that these complexes can effectively stimulate humoral immunity. They produce an intensity level of immunity that is four to eight times higher than that achieved with the standard adjuvant aluminum hydroxide. Additionally, the immunity generated was comparable to that formed with the iscommatrix containing Quill A.

Research has shown that these complexes can not only boost the levels of specific antibodies but also generate a high concentration of virus-neutralizing antibodies. This enhances the protective effectiveness of vaccine formulations that incorporate the studied adjuvant platform. Additionally, an investigation into the immunostimulating potential of this platform, using models of various genetically engineered proteins, demonstrated that the adjuvant can effectively stimulate a specific immune response when paired with different viral antigens.

## 4. Materials and Methods

### 4.1. Main Reagents

Vac-quil-5 QuilA adjuvant (InvivoGen, San Diego, CA, USA), vac-alu-50 Alhydrogel adjuvant, 2% (InvivoGen, San Diego, CA, USA), Yooo1905 Phosphatidylcholine from egg yolk (Merck, Darmstadt, Germany), D6277 N-Decanoyl_N-methyl glucamine (MEGA10) (Sigma-Aldrich, St. Louis, MO, USA), C3292 Cholesterol (Sigma-Aldrich, St. Lous, MO, USA) ab 98,703 goat anti mouse IgG2b (HRP) (Abcam Limited, Cambridge, UK), ab 98,693 goat anti mouse IgG1 (HRP) (Abcam Limited, Cambridge, UK), ab 97,245 goat anti mouse IgG2a (HRP) (Abcam Limited, Cambridge, UK), ab 97,023 goat anti mouse IgG (HRP) (Abcam Limited, Cambridge, UK), ab 97,230 goat anti mouse IgM (HRP) (Abcam Limited, Cambridge, UK), and ab 97,236 goat anti mouse IgA (HRP) (Abcam Limited, Cambridge, UK) were used.

### 4.2. Viruses and Antigens

Viruses (orthomyxoviruses: avian influenza virus strain A/Tern/South Africa/1/1961 (H5N3), highly pathogenic avian influenza virus strain A/FPV/Rostock/34 (H7N1), human influenza virus strain A/Almaty/8/98 (H3N2), and Tamiflu-resistant pandemic variant A/Vladivostok/2/09 (H1N1)) were obtained from the state collection of viruses at the Research and Production Center for Microbiology and Virology, Almaty, Kazakhstan. The virus cultivation, concentration, purification, and isolation of influenza virus glycoproteins from the purified concentrated viruses were described previously [40,41].

Commercially available antigens Herpes simplex virus-1 gD recombinant protein (where the *E. coli*-derived recombinant protein contains the HSV-1 gD immunodominant regions, 266–394 amino acids (HSV-221)), West Nile virus envelope recombinant protein (where the *E. coli*-derived recombinant protein contains the West-Nile N-terminus Envelope Virus immunodominant regions (WNV-001)), and Dengue virus subtypes 1 and 4 fused envelope 55 kDa recombinant protein (where the *E. coli*-derived recombinant 55kDa protein is a genetically engineered peptide derived from Dengue Type-1 and 4 to be expressed as a fused envelope: each part in this fusion contains 170 amino acids (positions 46–217)), and Dengue Type-1 and 4 were purified using a proprietary chromatographic technique (DEN-025)) and were purchased from ProSpec-Tany TechnoGene, Ltd., International, Ness-Ziona, Israel.

### 4.3. Preparation and Characterization of ISCOMATRIX

ISCOMATRIX was prepared via dialysis: Quil A–cholesterol–phospholipid of 1:1:1. Lipid (2 mg) and cholesterol (2 mg) were dissolved in 4% MEGA10 in a sterile tube. A total of 4 mL of PBS (0.01 M, pH 7.4) and 2 mg of Quil A were then added to the solution followed by 5 min sonication to facilitate dispersion. Solution was dialyzed against PBS for 16 h with twice the number of buffer changes. The ISCOMATRIX dispersion was subsequently extruded through (400, 200, 100) nm polycarbonate membranes (Avestin, Ottawa, ON, Canada) [42].

The zeta potentials and sizes of the formulations were measured using a Zetasizer (Nano-ZS, Malvern Instruments, Worcestershire, UK) [43]. Particle sizes were reported as the means ± standard deviations and polydispersity index (PDI) values (*n* = 3). Zeta potentials were reported as the means ± zeta deviations (*n* = 3). The structure of the ISCOMATRIX was confirmed via transmission electron microscopy (TEM) (30). Briefly, samples were coated onto glow-discharged, carbon-coated copper grids and negatively stained with 2% phosphotungstic acid (pH 5.2). After that, the samples were scanned using a Hitachi HT 7800 electron microscope (Hitachi High-Tech Corporation, Tokyo, Japan) with an acceleration voltage of 100 kV and a magnification of 100,000×.

The Encapsulation Efficiency of various nanoparticle formulations was determined through the centrifugation of the nanoparticle suspension at 19,000 rpm at a temperature of 12 °C for 30 min [44]. The amount of free hemagglutinins in the supernatant was measured via hemagglutination. The Encapsulation Efficiency (EE) of nanoparticles was calculated as follows: EE (%) = Total amount of hemagglutinin- amount of free hemagglutinin 100/Total amount of hemagglutinin. Each sample was measured in triplicate.

### 4.4. Cytotoxicity

The cytotoxicity of the compounds on Caco-2, HT29, MDCK, and macrophages was determined with the MTT test on 96-well microplates (Corning^®^, New York, NY, USA) with an area per well of 0.32 cm^2^ and 20,000 cells per well. The cytotoxicity evaluation was performed using a quantitative colorimetric MTT [3-(4,5-dimethylthiazol-2-yl)-2,5-diphenyl tetrazolium bromide, Sigma-Aldrich] assay [45]. The 50% cytotoxic concentration (CC50), which was defined as the quantity of sample resulting in 50% cell viability compared to control samples, was calculated.

### 4.5. Animals and Immunization

Specific pathogen-free male BALB/c mice, aged 4–5 weeks, were used in this study. The mice were acclimated under vivarium conditions for 2 weeks prior to the experiment. They were housed in individually ventilated cages (five mice per cage) under a 12 h light/dark cycle at a temperature of 20–25 °C. Standard rodent food and water were provided ad libitum. Each experimental group consisted of 5 animals.

All experimental procedures with mice were approved by the Research and Production Center for Microbiology and Virology Institutional Animal Care and Use Committee (conclusion of the bioethics commission dated 30 October 2023, N 02-09-188).

Animals were immunized using purified influenza virus glycoprotein antigens isolated from the purified concentrated influenza virus, viral proteins mixed with aluminum hydroxide, GPAs mixed with the nanocapsulated adjuvant from *Saponaria officinalis*, or GPAs mixed with ISCOM-Matrix. The animals were also immunized with commercial recombinant proteins or viral glycoproteins in a mixture with the studied adjuvants. In the experiments, the subcutaneous route of administration was used.

The doses of administered substances in μg per mouse were as follows: viral proteins—10; Saponin—15; ISCOM-Matrix—45; recombinant proteins—6; aluminum hydroxide—500 [46].

### 4.6. Enzyme-Linked Immunosorbent Assay (ELISA)

Serum samples were collected 3 weeks after immunization. Virus-specific antibodies were determined via ELISAs. The wells of the NUNC MaxiSorp plates (ThermoScientific, Waltham, MA, USA) were coated overnight at 4 °C without agitation and 100 µL per well with the researched viral antigen. During each incubation period, the plates were covered. After washing three times with 200 µL phosphate-buffered saline supplemented with 0.1% Tween-20 (PBS-T 0.1%) for five minutes, blocking was performed with 200 µL PBS-T (0.05%) with 0.2% (*w*/*v*) bovine serum albumin at room temperature for 2 h on a plate shaker. Serum samples were added in duplicate in serial 2-fold dilutions in 200 µL PBS-T (0.05%) with 10% (*w*/*v*) bovine serum albumin for 2 h and washed. The plate was treated with the secondary antibody (goat HRP anti-mouse antibody IgG, IgM, IgA, IgG1, IgG2a, IgG2b, IgG3). After another washing step, 5 mg of OPD (o-Phenylenediamine dihydrochloride) was diluted in 9 mL of distilled water and 1 mL of peroxide stable substrate. Then, 100 µL of this substrate solution was applied and incubated for 30 min in the dark. The addition of 100 µL of 2.5 M H_2_SO_4_ increased the measurement signal without enhancing the background. Therefore, absorbance measurements were performed at 490 nm using the spectrophotometer plate reader (Tecan Infinity M200) (Tecan Global Headquarters, Männedorf, Switzerland) [20,21,22].

### 4.7. Micro-Neutralization Assay

All micro-neutralization assays were performed with Madin–Darby canine kidney (MDCK) cells. Mouse sera were heat-inactivated (56 °C for 30 min) and stored at −20 °C until use. MDCK cells were seeded into flat-bottom 96-well plates (30,000 to 45,000 cells/well) to achieve confluent cell monolayers, which were used within 3 days of confluence. Two-fold serial dilutions of serum in MEM (Gibco, ThermoScientific, San Diego, CA, USA), starting at 1:10, were incubated with 50% tissue culture infective doses (TCID50) of virus for 2 h at 37 °C with 5% CO_2_. The serum/virus mixture was then added to MDCK cells in MEM (Gibco) supplemented with 10% heat-inactivated FCS and trypsin (Gibco). After 3 h at 37 °C with 5% CO_2_, the medium in each well was refreshed with MEM (Gibco). The cells were observed for the presence of cytopathic effect (3 to 5 days) and the micro-neutralization titer was defined as the highest dilution at which a confluent cell monolayer was maintained [47,48].

### 4.8. Statistical Analysis

The differences between the test sample and the untreated control were evaluated using Microsoft Excel 2016. The Microsoft Office suite was utilized for organizing and presenting the results in both tabular and graphical formats. All parameter values are expressed as means ± standard deviations (SDs). Significant differences between the experimental group and the control group were assessed using a two-tailed unpaired Student’s t-test and one-way analysis of variance (ANOVA). *p*-values less than 0.05 were considered statistically significant.

## 5. Conclusions

This study focused on saponins derived from Saponaria officinalis, which is considered one of the most promising plant sources for developing an adjuvant platform based on the formation of nanocomplexes. The capability of a nanoparticle adjuvant containing saponins from Saponaria officinalis to stimulate a humoral immune response was demonstrated using a model that included several viral proteins. Further research on these platforms could lead to a broader range of immunostimulants that enhance vaccine efficacy.

## Figures and Tables

**Table 1:** Particle size distribution, polydispersity index (PDI), zeta potential, and Encapsulation Efficiency of various supramolecular formulations.

Formulation	Size (nm)	PDI	Zeta Potential (mv)	Encapsulation Efficiency, %
ISCOMATRIX QuilA	75 ± 9.5	0.21 ± 0.08 *	−13.4 ± 5.6	92.34 ± 3.5
Complexes with saponins from *Saponaria officinalis*	70.2 ± 11.1	0.30 ± 0.05	−15.8 ± 5.2	94.2 ± 4.1

* Mean values and SDs from triplicate assays.

**Table 2:** Cytotoxicity of tested saponins in vitro.

Saponin	CC50 (μg/mL) Macrophages *	CC50 (μg/mL) MDCK *	CC50 (μg/mL) Caco2 *	CC50 (μg/mL) HT29 *
Quil-A	21.69 ± 3.5	28.13 ± 8.3	15.3 ± 2.8	18.25 ± 2.9
Saponin from *Saponaria officinalis*	161.00 ± 39.8	187.50 ± 33.1	142.24 ± 31.11	151.12 ± 28.23
Iscommatrix	65.00 ± 5.7	90.12 ± 7.9	100.13 ± 9.34	110.26 ± 8.67
Complexes with saponin from *Saponaria officinalis*	480.23 ± 32.25	570.36 ± 36.33	342.45 ± 34.52	456.47 ± 37.26

* Mean values and SDs from triplicate assays. Thus, in terms of its physicochemical characteristics, the saponin of *Saponaria officinalis* is comparable to Quil A and has less pronounced toxic properties.

## Data Availability

All the data generated or analyzed during this study have been included in the published article.

## References

[1] Hadian S.A., Rezayatmand R. (2022). Economic impact of acute respiratory disease pandemics: A scoping review. J. Res. Med. Sci..

[2] Hanage W.P., Schaffner W. (2025). Burden of Acute Respiratory Infections Caused by Influenza Virus, Respiratory Syncytial Virus, and SARS-CoV-2 with Consideration of Older Adults: A Narrative Review. Infect. Dis. Ther..

[3] Filipić B., Pantelić I., Nikolić I., Majhen D., Stojić-Vukanić Z., Savić S., Krajišnik D. (2023). Nanoparticle-Based Adjuvants and Delivery Systems for Modern Vaccines. Vaccines.

[4] Zhao T., Cai Y., Jiang Y., He X., Wei Y., Yu Y., Tian X. (2023). Vaccine adjuvants: Mechanisms and platforms. Signal Transduct. Target. Ther..

[5] Cui Y., Ho M., Hu Y., Shi Y. (2024). Vaccine adjuvants: Current status, research and development, licensing, and future opportunities. J. Mater. Chem. B..

[6] Korchowiec B., Korchowiec J., Kwiecińska K., Gevrenova R., Bouguet-Bonnet S., Deng C., Henry M., Rogalska E. (2022). The Molecular Bases of the Interaction between a Saponin from the Roots of Gypsophila paniculata L. and Model. Lipid Membranes. Int. J. Mol. Sci..

[7] Eygeris Y., Jozic A., Henderson M.I., Nelson D., Sahay G. (2025). Exploring the potential of saponins as adjuvants in lipid-nanoparticle-based mRNA vaccines. Mol. Ther. Methods Clin. Dev..

[8] Magedans Y.V., Yendo A.C., Costa F., Gosmann G., Fett-Neto A.G. (2019). Foamy matters: An update on Quillaja saponins and their use as immunoadjuvants. Future Med. Chem..

[9] Sun H.X., Xie Y., Ye Y.P. (2009). ISCOMs and ISCOMATRIX. Vaccine.

[10] Osterhaus A.D., Rimmelzwaan G.F. (1998). Induction of virus-specific immunity by iscoms. Dev. Biol. Stand..

[11] Petkov V., Tsibranska S., Manoylov I., Kechidzhieva L., Ilieva K., Bradyanova S., Ralchev N., Mihaylova N., Denkov N., Tchorbanov A. (2025). ISCOM-type matrix from beta-escin and glycyrrhizin saponins. Heliyon.

[12] Stertman L., Palm A.E., Zarnegar B., Carow B., Lunderius Andersson C., Magnusson S.E., Carnrot C., Shinde V., Smith G., Glenn G. (2023). The Matrix-M™ adjuvant: A critical component of vaccines for the 21st century. Hum. Vaccin. Immunother..

[13] Fries L., Cho I., Krähling V., Fehling S.K., Strecker T., Becker S., Hooper J.W., Kwilas S.A., Agrawal S., Wen J. (2020). Randomized, blinded, dose-ranging trial of an ebola virus glycoprotein nanoparticle vaccine with Matrix-M adjuvant in healthy adults. J. Infect. Dis..

[14] Datoo M.S., Natama M.H., Somé A., Traoré O., Rouamba T., Bellamy D., Yameogo P., Valia D., Tegneri M., Ouedraogo F. (2021). Efficacy of a low-dose candidate malaria vaccine, R21 in adjuvant Matrix-M.; with seasonal administration to children in Burkina Faso: A randomised controlled trial. Lancet.

[15] Shinde V., Cai R., Plested J., Cho I., Fiske J., Pham X., Zhu M., Cloney-Clark S., Wang N., Zhou H. (2021). Induction of cross-reactive hemagglutination inhibiting antibody and polyfunctional CD4+ T-cell responses by a recombinant matrix-M-adjuvanted hemagglutinin nanoparticle influenza vaccine. Clin. Infect. Dis..

[16] Shinde V., Cho I., Plested J.S., Agrawal S., Fiske J., Cai R., Zhou H., Pham X., Zhu M., Cloney-Clark S. (2022). Comparison of the safety and immunogenicity of a novel Matrix-M-adjuvanted nanoparticle influenza vaccine with a quadrivalent seasonal influenza vaccine in older adults: A phase 3 randomised controlled trial. Lancet Infect. Dis..

[17] Huis in’t Veld L.G., Cornelissen L.A., van den Bogaard L., Ansems M., Ho N.I., Adema G.J. (2025). Saponin-based adjuvant uptake and induction of antigen cross-presentation by CD11b+ dendritic cells and macrophages. NPJ Vaccines.

[18] Pulendran B., Arunachalam P.S., O’Hagan D.T. (2021). Emerging concepts in the science of vaccine adjuvants. Nat. Rev. Drug Discov..

[19] Ou B.S., Baillet J., Filsinger Interrante M.V., Adamska J.Z., Zhou X., Saouaf O.M., Yan J., Klich J.H., Jons C.K., Meany E.L. (2024). Saponin nanoparticle adjuvants incorporating Toll-like receptor agonists drive distinct immune signatures and potent vaccine responses. Sci Adv..

[20] Berezin V.E., Bogoyavlenskiy A.P., Tolmacheva V.P., Makhmudova N.R., Khudyakova S.S., Levandovskaya S.V., Omirtaeva E.S., Zaitceva I.A., Tustikbaeva G.B., Ermakova O.S. (2008). Immunostimulating complexes incorporating Eimeria tenella antigens and plant saponins as effective delivery system for coccidia vaccine immunization. J. Parasitol..

[21] Turmagambetova A.S., Alexyuk P.G., Bogoyavlenskiy A.P., Zaitseva I.A., Omirtaeva E.S., Alexyuk M.S., Sokolova N.S., Berezin V.E. (2017). Adjuvant activity of saponins from Kazakhstani plants on the immune responses to subunit influenza vaccine. Arch. Virol..

[22] Alexyuk P.G., Bogoyavlenskiy A.P., Alexyuk M.S., Turmagambetova A.S., Zaitseva I.A., Omirtaeva E.S., Berezin V. (2019). Adjuvant activity of multimolecular complexes based on Glycyrrhiza glabra saponins, lipids, and influenza virus glycoproteins. Arch. Virol..

[23] Mamo T., Poland G.A. (2012). Nanovaccinology: The next generation of vaccines meets 21st century materials science and engineering. Vaccine.

[24] Sadr S., Poorjafari Jafroodi P., Haratizadeh M.J., Ghasemi Z., Borji H., Hajjafari A. (2023). Current status of nano-vaccinology in veterinary medicine science. Vet. Med. Sci..

[25] Yasamineh S., Kalajahi H.G., Yasamineh P., Yazdani Y., Gholizadeh O., Tabatabaie R., Afkhami H., Davodabadi F., Farkhad A.K., Pahlevan D. (2022). An overview on nanoparticle-based strategies to fight viral infections with a focus on COVID-19. J. Nanobiotechnol..

[26] Zaheer T., Pal K., Zaheer I. (2021). Topical review on nano-vaccinology: Biochemical promises and key challenges. Process Biochem..

[27] Li X., Sloat B.R., Yanasarn N., Cui Z. (2011). Relationship between the size of nanoparticles and their adjuvant activity: Data from a study with an improved experimental design. Eur. J. Pharm. Biopharm..

[28] Bo C., Wei X., Wang X., Ji W., Yang H., Zhao Y., Wang H. (2023). Physicochemical properties and adsorption state of aluminum adjuvants with different processes in vaccines. Heliyon.

[29] Zhong L., Fu S., Peng X., Zhan H., and Sun R. (2012). Colloidal stability of negatively charged cellulose nanocrystalline in aqueous systems. Carbohydr. Polym..

[30] Cao J., Zhang X., Wu X., Wang S., Lu C. (2016). Cellulose nanocrystals mediated assembly of graphene in rubber composites for chemical sensing applications. Carbohydr. Polym..

[31] Silveira F., Cibulski S.P., Varela A.P., Marqués J.M., Chabalgoity A., de Costa F., Yendo A.C., Gosmann G., Roehe P.M., Fernández C. (2011). Quillaja brasiliensis saponins are less toxic than Quil A and have similar properties when used as an adjuvant for a viral antigen preparation. Vaccine.

[32] Tucker I.M., Burley A., Petkova R.E., Hosking S.L., Webster J.R.P., Li P.X., Ma K., Doutch J., Penfold J., Thomas R.K. (2021). Self-assembly in saponin mixtures: Escin / tea, tea / glycyrrhizic acid, and escin / glycyrrhizic acid mixtures. Colloids Surf. A.

[33] Wang D., Sha L., Xu C., Huang Y., Tang C., Xu T., Li X., Di D., Liu J., Yang L. (2022). Natural saponin and cholesterol assembled nanostructures as the promising delivery method for saponin. Colloids Surf. B Biointerfaces.

[34] Chung K.Y., Coyle E.M., Jani D., King L.R., Bhardwaj R., Fries L., Smith G., Glenn G., Golding H., Khurana S. (2015). ISCOMATRIX™ adjuvant promotes epitope spreading and antibody affinity maturation of influenza A H7N9 virus like particle vaccine that correlate with virus neutralization in humans. Vaccine.

[35] Hägglund S., Hu K., Blodörn K., Makabi-Panzu B., Gaillard A.L., Ellencrona K., Chevret D., Hellman L., Bengtsson K.L., Riffault S. (2014). Characterization of an experimental vaccine for bovine respiratory syncytial virus. Clin. Vaccine Immunol..

[36] Fossum C., Hjertner B., Ahlberg V., Charerntantanakul W., McIntosh K., Fuxler L., Balagunaseelan N., Wallgren P., Bengtsson K.L. (2014). Early inflammatory response to the saponin adjuvant Matrix-M in the pig. Vet. Immunol. Immunopathol..

[37] Oda K., Matsuda H., Murakami T., Katayama S., Ohgitani T., Yoshikawa M. (2000). Adjuvant and haemolytic activities of 47 saponins derived from medicinal and food plants. Biol. Chem..

[38] Ojiako C.M., Okoye E.I., Oli A.N., Ike C.J., Esimone C.O., Attama A.A. (2019). Preliminary studies on the formulation of immune stimulating complexes using saponin from Carica papaya leaves. Heliyon.

[39] Wang P., Ding X., Kim H., Michalek S.M., Zhang P. (2020). Structural Effect on Adjuvanticity of Saponins. J. Med. Chem..

[40] Klimov A., Balish A., Veguilla V., Sun H., Schifferet J., Lu X., Katz J.M., Hancock K. (2012). Influenza virus titration, antigenic characterization, and serological methods for antibody detection. Methods Mol. Biol..

[41] Carlsson N., Borde A., Wölfel S., Åkerman B., Larsson A. (2011). Quantification of protein concentration by the Bradford method in the presence of pharmaceutical polymers. Anal. Biochem..

[42] Golali E., Jaafari M.R., Khamesipour A., Abbasi A., Saberi Z., Badiee A. (2012). Comparison of in vivo Adjuvanticity of Liposomal PO CpG ODN with Liposomal PS CpG ODN:Soluble Leishmania Antigens as a Model. Iran. J. Basic. Med. Sci..

[43] Jaafari M.R., Ghafarian A., Farrokh-Gisour A., Samiei A., Kheiri M.T., Mahboudi F., Barkhordari F., Khamesipour A., McMaster W.R. (2006). Immune response and protection assay of recombinant major surface glycoprotein of leishmania (rgp63) reconstituted with liposomes in BALB/c mice. Vaccine.

[44] Wadhwa S., Paliwal R., Paliwal S.R., Vyas S.P. (2010). Hyaluronic acid modified chitosan nanoparticles for effective management of glaucoma: Development, characterization, and evaluation. J. Drug Target..

[45] Stepanenko A.A., Dmitrenko V.V. (2015). Pitfalls of the MTT assay:direct and off-target effects of inhibitors can result in over/underestimation of cell viability. Gene.

[46] He P., Zou Y., Hu Z. (2015). Advances in aluminium hydroxide-based adjuvant research and its mechanism. Hum. Vaccin. Immunother..

[47] Berezin V.E., Zaides V.M., Isaeva E.S., Artamonov A.F., Zhdanov V.M. (1988). Controlled organization of multimolecular complexes of enveloped virus glycoproteins: Study of immunogenicity. Vaccine.

[48] Verschoor C.P., Singh P., Russell M.L., Bowdish D.M., Brewer A., Cyr L., Ward B.J., Loeb M. (2016). Correction: Microneutralization assay titres correlate with protection against seasonal influenza H1N1 and H3N2 in children. PLoS ONE.

